# Human Nail Clippings as a Source of DNA for Genetic Studies

**DOI:** 10.4236/ojepi.2015.51006

**Published:** 2015-02-01

**Authors:** Le Truong, Hannah Lui Park, Seong Sil Chang, Argyrios Ziogas, Susan L. Neuhausen, Sophia S. Wang, Leslie Bernstein, Hoda Anton-Culver

**Affiliations:** 1 Department of Epidemiology, Genetic Epidemiology Research Institute, University of California, Irvine, CA, USA; 2 Division of Cancer Etiology, Department of Population Sciences, Beckman Research Institute, City of Hope, Duarte, CA, USA

**Keywords:** Single Nucleotide Polymorphism (SNP), Nail Clippings, Genotyping, Whole Genome Amplification (WGA)

## Abstract

Blood samples have traditionally been used as the main source of DNA for genetic analysis. However, this source can be difficult in terms of collection, transportation, and long-term storage. In this study, we investigated whether human nail clippings could be used as a source of DNA for SNP genotyping, null-allele detection, and whole-genome amplification. From extracted nail DNA, we achieved amplicons up to a length of ~400 bp and >96% concordance for SNP genotyping and 100% concordance for null-allele detection compared to DNA derived from matched blood samples. For whole-genome amplification, OmniPlex performed better than Multiple Displacement Amplification with a success rate of 89.3% and 76.8% for SNP genotyping and null-allele detection, respectively. Concordance was ~98% for both methods. When combined with OmniPlex whole-genome amplification, human nail clippings could potentially be used as an alternative to whole blood as a less invasive and more convenient source of DNA for genotyping studies.

## 1. Introduction

Researchers have traditionally used blood as the source of DNA to investigate genetic factors in epidemiologic studies. However, with improved analytic methodologies reducing the amount of DNA required, less invasive sources of DNA with lower yield, including buccal (cheek) cells, urine, stool, saliva, chewing gum, hair, and nails, have gained the interest of many investigators. Like buccal swabs and saliva, nail collection has many benefits: it is non-invasive, simple, and inexpensive to transport by normal postal mail, as opposed to whole blood and many other DNA sources. While other sources of DNA may require kits or specialized personnel for sample collection, nail samples are self-collectable in a small envelope and can be stored long term at room temperature before processing. Although buccal swabs and saliva samples were demonstrated to be stable at room temperature for over one and five years, respectively [1] [2], nail DNA was shown to be successful in SNP detection assays even when clippings were stored at room temperature for over 20 years [3]. 

Large epidemiological studies require a high-throughput, low-cost and reliable method for genomic DNA (gDNA) collection. However, higher amounts of DNA may be required for some types of analyses, as is the case for SNP linkage panels. In the past decade, two whole genome amplification (WGA) methods were described in which DNA was successfully amplified from a very small amount of gDNA [4] [5] and exhibited excellent SNP genotype performance [6] [7]. The first is multiple-displacement amplification (MDA), which is based on strand-displacement synthesis using the highly processive ϖ29 DNA polymerase and random exonuclease-resistant primers in an isothermal amplification reaction [4]. MDA is modified from a rolling circle amplification method that was designed to amplify large circular DNA [8] and has the capability of generating DNA products of >10 kb in length. The second method, OmniPlex, was introduced by Rubicon Genomics (www.rubicongenomics.com) and relies upon high-fidelity DNA polymerase to generate a library of DNA fragments of approximately 100 bp to 1000 bp from randomly fragmented gDNA [9]. DNA products from OmniPlex WGA were reported to be reamplifiable to achieve over a million-fold DNA without degradation.

The goal of this study was to do a pilot analysis on the feasibility of using a very small amount of human nail clippings for genotyping. Our aims were to determine the amount and purity of total DNA and dsDNA from nail clippings, to assess the maximal amplicon size for PCR, and to determine the genotyping success rate and concordance before and after MDA and OmniPlex WGA in comparison to DNA extracted from whole blood.

## 2. Methods

### 2.1. Sample Collection and DNA Extraction

For this small pilot study, we obtained 14 individual sets of nail samples from California Teachers Study (CTS) participants who had previously also donated blood [10]. DNA extracted from their blood samples was used for comparison to nail-extracted DNA. Participants were asked to donate nail clippings from as many fingers and/or toes as possible. Samples were self-collected, packed in a small envelope, and sent by participants via regular postal mail to our laboratory. Fifty to 200 mg of nails were collected from each participant, and 4 – 10 mg (less than one small nail) were used for DNA extraction. If the presence of nail polish was detected (7 out of 14 samples), clippings were washed in acetone before extraction. DNA was extracted using QIAamp MiniElute columns (Qiagen, Inc., Valencia, CA, USA) according to the manufacturer’s protocol. Nail clippings were finely cut by a pair of small scissors before overnight digestion in proteinase K. Extracted DNA was stored at 4°C for further analysis. 

### 2.2. DNA Quantification

Nail-extracted DNA was quantified by a NanoDrop-1000 spectrophotometer (NanoDrop Products, Wilmington, DE) and the purity of DNA was assessed by calculating the 260/280 and 260/230 absorbance ratios. Double-stranded DNA concentration was measured by a PicoGreen quantification kit (Quant-it™ dsDNA HS assay kit, Invitrogen, Carlsbad, CA). 

### 2.3. PCR and Electrophoresis

To determine the optimal amplicon size of nail DNA, primers were designed to amplify fragments of a nuclear-encoded gene [*breast cancer* 1, *early onset* (*BRCA*1)] and a mitochondrial-encoded gene [*cytochrome c oxidase I* (*COI*)] with amplicons ranging from 94 bp to 918 bp, as listed in Table 1. PCR was carried out in a total volume of 50 ul containing 10 ng of DNA and a final concentration of 0.4 pM of each primer, 1mM of dNTP mixture (Epicentre Technologies, Madison, WI), 3 mM of MgCl_2_, and 1 unit of Taq DNA polymerase (Qiagen). Amplification conditions consisted of an initial denaturing step at 95°C for 3 min followed by 35 cycles of 95°C for 50 sec, 56°C for 50 sec, 72°C for 1 min. Amplification concluded with a terminal extension step at 72°C for 10 min. A portion (15 ul) of the PCR product was loaded onto a 3% Metaphor agarose gel (BMA, Rockland, ME) and visualized under UV light after staining with ethidium bromide. 

### 2.4. DNA Amplification

MDA (Qiagen Repli GU trafast Kit) and OmniPlex (Sigma GenonePlex Complete WGA Kit) were carried out according to the manufacturers’ protocols. Each MDA and OmniPlex WGA contained 20 ng of DNA in a 25 ul and 75 ul reaction, respectively. A portion of amplified product was loaded on a 2% TBE agarose gel for visualization, and the remaining was diluted by 1/25 for downstream applications. All 14 samples were amplified with a negative control. OmniPlex WGA also had a positive control which was included with the Sigma Genoplex Complete WGA kit. 

### 2.5. SNP Detection Assays

Four SNP primer/probe sets were used in this study (Table 2), two of which were custom-designed [Assays-by-Design for *excision repair cross-complementing rodent repair deficiency*, *complementation group* 2 (*ERCC*2) and *transforming growth factor*, *beta* 1 (*TGFb*1)] and two of which were pre-designed [Assays-On-Demand for *N-acetyltransferase* 2 (*NAT*2) and *catechol-O-methyltransferase* (*COMT*)] by ABI (Applied Biosystems, Inc., Foster City, CA). Reactions were carried out in a total volume of 5ul containing 7.5 ng of non-amplified DNA template (as measured by NanoDrop) or 1 ul of 1/25 dilution of amplified products. Each reaction had a final concentration of 1x primer/probe mix, 3 mM MgCl_2_, and 1x TaqMan Universal Master Mix (ABI). Real-time PCRs were performed in an ABI PRISM 7900HT with the following conditions: 50°C for 2 min, 95°C for 10 min, followed by 40 cycles of 95°C for 15 sec and 60°C for 1min. SNP analysis was done using the installed SDS Enterprise software. 

### 2.6. Null-Allele Detection Assays

The ABI TaqMan copy number assay for *glutathione-S-transferase-M*1 (*GSTM*1) (Hs02575461_cn) was used with an ABI copy number reference assay in a duplex real-time PCR reaction. Each reaction contained either 7.5 ng of non-amplified DNA or 1 ul of 1/25 dilution of WGA product in a total volume of 10 ul, including reagents as published in the ABI protocol. As a standard, each whole blood DNA sample had four replicates, whereas other samples were each analyzed in duplicate. All reactions were run together on a single 384-well microplate, and the same real-time PCR parameters were used as in the SNP genotyping reactions described above. Null-allele deletion analysis was performed using ABI CopyCaller software. 

## 3. Results

### 3.1. DNA Yield

On average, 6.3 milligrams of nail were used for DNA extraction, yielding an average of 544 ng DNA or 91.7 ng/mg of nail as measured by NanoDrop (Table 3). However, the average yields were 254 ng DNA or 43.2 ng/mg of nail as measured by PicoGreen. The PicoGreen:NanoDrop ratios ranged from 0.194 to 0.801 with an average of 0.476, suggesting the presence of a high concentration of single-stranded DNA or RNA in many samples. The 260/280 absorbance ratios ranged from 1.04 to 1.86 with an average of 1.43, and the 260/230 absorbance ratios ranged from 0.53 to 5.13, indicating the presence of contaminants such as proteins, phenol, and/or other substances in a number of samples (Table 3). 

### 3.2. PCR and Electrophoresis

**Figure 1:**
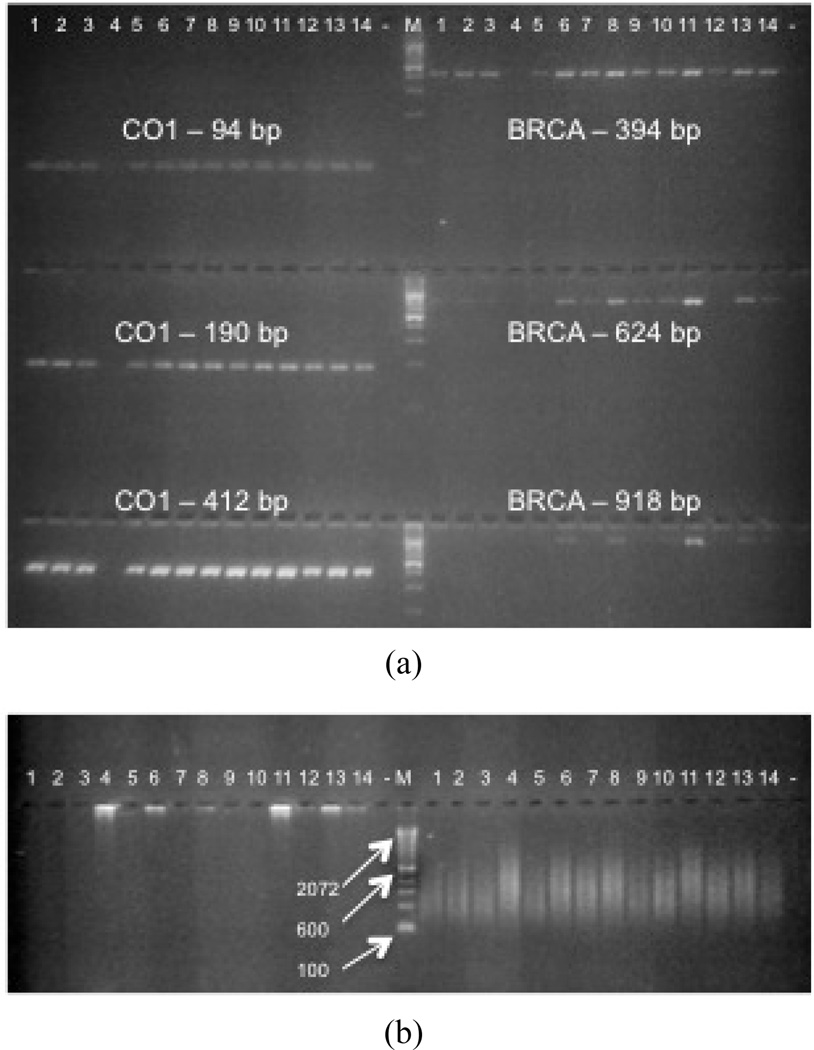
(a) PCR products of different amplicon sizes for *CO*1 and *BRCA*1. All 14 samples and negative controls (−) were loaded onto a 3% TBE agarose gel pre-stained with ethidium bromide, separated by electrophoresis, and viewed under UV light. M: 100 bp molecular ladder. (b) WGA products on 2% TBE agarose pre-stained with ethidium bromide, separated by electrophoresis, and viewed under UV light. M: 100bp molecular ladder.

All nail samples were successfully amplified by single-fragment PCR for both *COI* and *BRCA*1 when the amplicon sizes were up to ~400 bp (Figure 1(a)). Sample 4 showed weak signals for all reactions despite a high PicoGreen: NanoDrop ratio and 260/280 UV absorbance ratio of 1.61. However, its 260/230 UV absorbance ratio was 5.13, suggesting that a high salt concentration may have hindered the amplification reaction. The success rate decreased to 79% (11/14) when amplicon size was increased to 600 bp and was poorest at 900 bp, with only 50% (7/14) of samples being successfully amplified. 

### 3.3. Whole Genome Amplification

MDA utilizes random primers and ϖ29 DNA polymerase in a strand-displacement reaction, forming fragment sizes of >10 kb, whereas OmniPlex amplifies shorter DNA fragments from ~100 bp to 1kb. 20% (5 ul) and 10% (7.5 ul) of MDA and OmniPlex WGA products, respectively, were loaded side-by-side and run on a 2% agarose gel (Figure 1(b)). MDA products hardly migrated due to their large fragment sizes, and there was large variation in the extent of amplification among samples, with some being easily visible and others not so. The intensity of staining of each MDA product was not correlated with the concentration or purity of starting material. OmniPlex, on the other hand, showed consistently strong signals between 100 and 1000 bp. Sample 4 showed strong signals after WGA, confirming the presence of DNA despite exhibiting weak amplification in the initial PCR reaction. 

### 3.4. Pre-WGA SNP Genotyping

**Figure 2:**
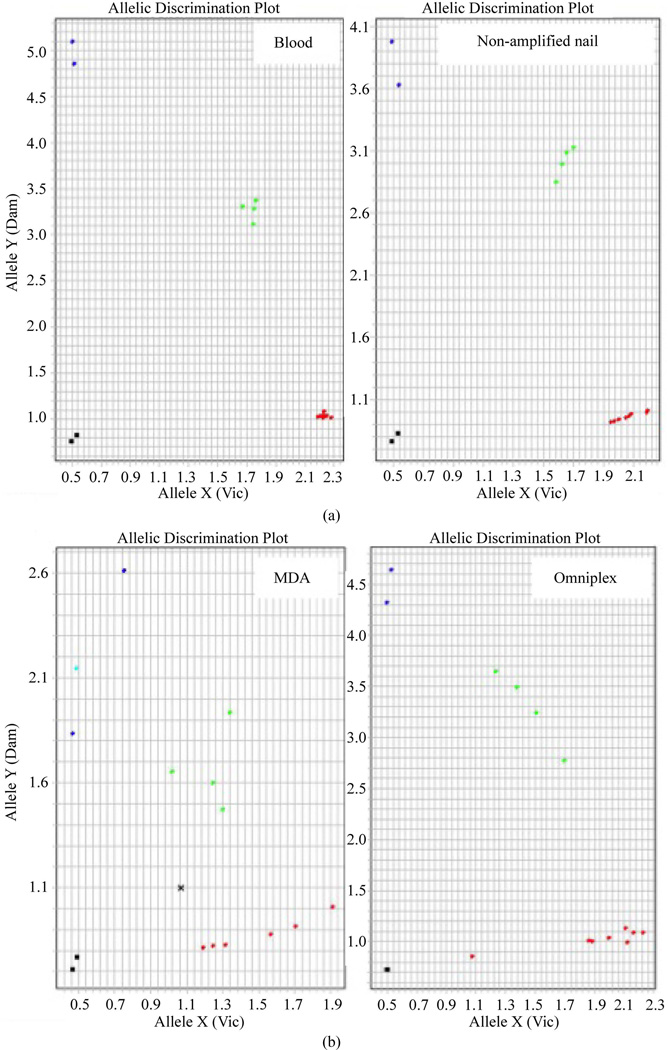
(a) *ERCC*2, 751A/C, pre-WGA SNP genotyping represents the general allelic discriminating plots for DNA from blood (left) and from matched, non-amplified nail clippings (right). Samples from blood generally clustered more tightly. Allele X (red): homozygous wild-type (AA); Allele Y (blue): homozygous variant (CC); Both (green): heterozygous (AC); NTC (black): Non-Template Control. (b) *ERCC*2, 751A/C, post-WGA SNP genotyping represents the general allelic discriminating plots for nail-extracted DNA samples amplified by MDA (left) and Omniplex (right). Amplified nail-extracted DNA samples generally scattered more on the graphs compared to non-amplified samples. Left: post-MDA with one undetermined (x) and one inconsistency (#2). Right: post-OmniPlex. Allele X (red): homozygous wild-type (AA); Allele Y (blue): homozygous variant (CC); Both (green): heterozygous (AC); NTC (black): Non-Template Control.

In general, SNP genotyping of non-amplified nail-extracted DNA showed good clustering although it was it was not as tight as compared to blood-extracted DNA (Figure 2(a)), likely due to variation in concentrations of double-stranded DNA and higher level of contaminants in nail-extracted DNA. All SNP genotyping was successful in non-amplified nail-extracted DNA except that *TGFβ* and *COMT* were both read as “undetermined” for sample 4, the same sample that exhibited poor PCR amplification of *COI* and *BRCA*1 (Figure 1(a)). For *TGFβ* and *COMT*, a mismatch was also observed for each in 1 out of the 13 successful assays (sample 1). Otherwise, nail-extracted DNA showed perfect concordance with matched blood-extracted DNA for all 4 SNP assays. The mean concordance rate was 96.2% (Table 3). 

### 3.5. Post-WGA SNP Genotyping

Post-WGA SNP genotyping showed even more scattering of samples, most likely from even larger variations in concentration of the DNA templates, since amplified samples were diluted but not quantified prior to genotyping (Figure 2(b)). Samples amplified by MDA exhibited a lower success rate (mean 76.8%) than non-amplified samples (mean 96.5%), but with only one discordance in *ERCC*2 SNP genotyping. OmniPlex had a higher success rate (mean 89.3%), but it also had one discordance, in *COMT* SNP (Table 4). Compared to non-amplified DNA, DNA from both WGA methods exhibited comparable concordance of genotype with blood-extracted DNA (~98%). 

### 3.6. Null Deletion Assay: GSTM1

**Figure 3:**
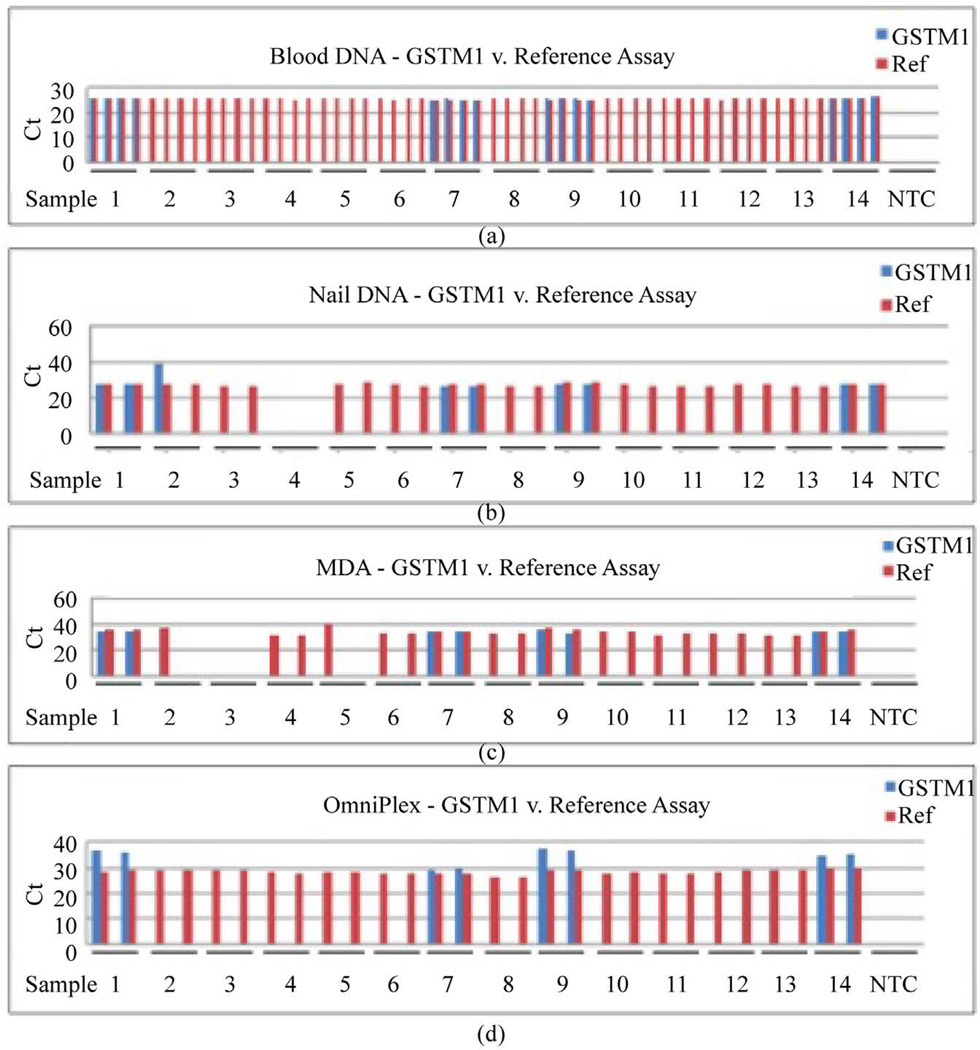
*GSTM*1 null-allele detection and reference assays in real-time PCR reactions. (a) Quadruplicate reactions of whole blood showed 4 samples with *GSTM*1 alleles (#1, 7, 9, 14). (b) Both duplicates of non-amplified nail DNA showed no product for sample #4 and inconsistency in the duplicates for sample #2. (c) Duplicate reactions of nail DNA post-MDA WGA showed failed amplification in both duplicates for sample #3, whereas samples #2 and #5 each had one failed duplicate. (d) Duplicates of nail DNA post-Omni- Plex WGA with complete consistency in all 14 samples.

Of the 14 whole blood samples tested, 10 samples were null for *GSTM*1, with all 10 replicates showing excellent consistency across the 14 samples (Figure 3(a)). No-template control (NTC) showed zero activity for both reference and *GSTM*1 assays. Non-amplified nail-extracted DNA correctly identified the null allele (Figure 3(b)) except that one sample failed (sample 4) and another one was inconsistent between duplicates (sample 2). The null alleles of *GSTM*1 were also correctly identified in samples amplified by MDA or OmniPlex except for three MDA-amplified samples (samples 2, 3, and 5) in which the assay failed (Figure 3(c) and Figure 3(d)). Overall, all nail-extracted DNA samples, amplified or not, had an excellent concordance rate (100%) for null-allele detection, but OmniPlex had the best success rate (100%) (Table 4). 

## 4. Discussion

Traditionally, researchers in epidemiology use blood as a source of DNA for molecular genetic studies. However, this biospecimen can be extremely costly and difficult in terms of collection, transportation, and long-term storage. With improved techniques that require less DNA, it has become possible to use other sources of DNA; however, potential DNA sources must be of acceptable quality and quantity, free of representational bias and discrepancy in comparison to blood DNA. One study has shown that nail-extracted DNA quality was reported to be consistent even when nail clippings stored at room temperature for over 20 years, and its quantity was sufficient for a multiplexed SNP detection assay [3]. In that study, amplicons of ≤596 bp could be amplified and the genotype concordance was above 96%. We further examined the quantity, quality, purity, and the capability of DNA extracted from a very small amount of human nail clippings in generating a large amplicon. We also assessed the potential bias of nail-extracted DNA in SNP genotyping and null-allele detection before and after amplification by two different WGA methods. We then compared the percentage of successful calls and concordance between unamplified and amplified nail-extracted DNA to blood-extracted DNA from the same participants. 

On average, approximately 90 ng of total DNA was extracted per milligram of nail clippings, which is sufficient for more than 10 SNP genotype reactions (5 – 10 ng of DNA template per reaction). The low 260/280 and 260/230 absorbance ratios in some of the samples indicated the presence of contaminants such as keratin, other proteins, and nail polish, as well as DNA extraction components such as EDTA and guanidine HCl, which may have required additional purification processing post-DNA isolation. Notably, the one sample which was unsuccessful in two SNP assays and the null-allele assay (sample 4) was the one with the highest 260/230 absorbance ratio, suggesting that high concentration of a contaminant may have hindered these genotyping reactions. However, after WGA, success rates were comparable to those of the other samples, and concordance was 100% among successful assays. These findings suggest that a purification step after DNA isolation from nail clippings may increase the success rate of genotyping assays. 

Comparison of DNA quantitation by PicoGreen and NanoDrop indicated that double-stranded DNA was at a concentration about half the total DNA concentration, suggesting a high concentration of single-stranded DNA, most likely primers. However, we were able to amplify amplicons up to 400 bp with a >96% success rate, suggesting that this method is suitable for SNP genotyping since most assays amplify regions that are 250 bp or smaller. 

WGA is necessary to generate enough templates for large-scale epidemiology genetic studies because nails can only provide a limited source of DNA. Previous studies have reported high concordance (>99.8%) for both MDA and OmniPlex [6] [11] for gDNA from lymphocytes. Another study reported that MDA performed better than OmniPlex for DNA samples from whole blood and buccal swabs [12]. Our study, however, showed that OmniPlex outperformed MDA in both success rate and reliability for genotyping of DNA from nails. The poor performance of MDA was likely attributed to its non-uniform amplification, and might be improved by modifying the manufacture protocol, such as increasing the amplification time from 1.5 hour to 16 or 18 hours as suggested by previous studies [4] [6]. OmniPlex showed uniform DNA fragments of 100 bp – 1000 bp across all samples tested with a slightly lower SNP genotyping success rate but comparable concordance with matched non-amplified samples. OmniPlex WGA achieved 100% in both success rate and concordance for null-allele detection. In a 75 ul reaction, an OmniPlex-amplified product originating from less than one nail clipping can provide enough template DNA for over 1800 individual SNP genotype reactions (25× dilution). 

## 5. Conclusion

This pilot study demonstrates that nails can potentially serve as a source of DNA for molecular genetic studies. Nail clippings have many advantages including non-invasiveness collection, self-collectability without requiring a tissue collecting kit or assistance by research staff, simplicity and low-cost to transport by normal postal mail, and ability to be stored long-term at room temperature. However, extraction of DNA from nail clippings is a more laborious process than from other tissue sources and yields DNA with relatively high levels of contaminants which may affect downstream amplification and analysis. While nail clippings provide an alternative source of DNA for SNP genotyping and null-allele detection assays, we observed a lower genotyping success rate (92.7% for 2 genes) than that which we typically observe with DNA from blood or saliva. In addition, due to fragmentation, nail DNA is unsuitable if long PCR amplicons are desired. Based on our preliminary results, nail clippings are a feasible alternative source of DNA but with limitations in performance, and further efforts in validating the use of nails for multiplex genotyping assays are warranted. 

## Figures and Tables

**Table 1:** Primer sets and target amplicon sizes for *CO*1 and *BRCA*1.

Gene	Common forward primer	Reverse primers	Amplicon size (bp)
		TGTGCCTAGGACTCCAGCTC	94
*CO*1	TTCGCCGACCGTTGACTATTCTCT	TTACAAATGCATGGGCTGTG	190
		GGTGGGAGTAGTTCCCTGCT	412
		CCCATCTGTTATGTTGGCTC	394
*BRCA*1	TAGCCAGTTGGTTGATTTCC	TGATTCAGACTCCCCATCAT	624
		GACGCTCTTGTATTATCTGTGGCTC	918

**Table 2:** Primers and probes for SNP/copy number detection.

	Forward primer	Reverse primer
Gene, SNP		
*ERCC*2, 751A/C^a^	GCCTGGAGCAGCTAGAATCAG	ACCCGCCCCACTCAGA
*TGFb*1, −509C/T^b^	GCCTCCGGAGGGTGTCA	AAGGAGAGCAATTCTTACAGGTGTCT
*NAT*2, 590G/A	ABI Assays-on-Demand: C 1204091 10	
*COMT*, 1158C/G	ABI Assays-on-Demand: C 2538750 10	
Gene, copy # *GSTM*1	ABI Assay ID: Hs02575461_cn	

a Vic probe: CTCTATCCTCTTCAGCGTC; Fam probe: TATCCTCTGCAGCGTC;

b Vic probe: CCATCCCTCAGGTGT; Fam probe: CATCCTTCAGGTGTC.

**Table 3:** DNA yields from nail clippings as measured by two methods, NanoDrop and PicoGreen, and UV absorbance ratios.

Nail Sample #	Amt, nail used (mg)	DNA yield (ng), as measured by NanoDrop	DNA yield (ng/mg nail), as measured by NanoDrop	DNA yield (ng), as measured by PicoGreen	DNA yield (ng/mg nail), as measured by PicoGreen	PicoGreen: NanoDrop ratio	260/280 absorbance ratio	260/230 absorbance ratio
1	4.2	521	124	160	38.1	0.307	1.04	0.7
2	4.6	543	118	194	42.2	0.357	1.16	0.8
3	4.6	685	149	381	82.9	0.556	1.38	0.74
4	5.6	535	95.5	429	76.5	0.801	1.61	5.13
5	5.1	576	113	112	21.9	0.194	1.37	0.72
6	6.7	412	61.5	202	30.1	0.490	1.86	0.59
7	9.1	474	52.1	210	23.0	0.442	1.08	3.28
8	9.5	842	88.6	550	57.9	0.653	1.66	1.04
9	5.5	495	90.0	199	36.2	0.402	1.37	0.65
10	9.7	428	44.1	170	17.6	0.398	1.44	0.53
11	7.0	702	100	427	61.0	0.608	1.69	0.86
12	5.8	349	60.2	166	28.6	0.474	1.49	0.58
13	6.1	516	84.6	335	54.9	0.649	1.76	0.95
14	5.2	533	103	180	34.5	0.337	1.11	0.79
Mean	6.3	544	91.7	265	43.2	0.476	1.43	1.24
SD	1.9	128	29.7	133	20.4	0.163	0.265	1.32

**Table 4:** Summary of success^a^ and concordance^b^ rates in SNP genotyping and null-allele detection of nail-extracted DNA compared to DNA from matched blood samples.

	Non-amplified nail		Amplified by MDA		Amplified by OmniPlex	

Genes	Success (%)	Concordance (%)	Success (%)	Concordance (%)	Success (%)	Concordance (%)
*ERCC*2	100	100	92.9	92.3	100	100
*NAT*2	100	100	64.3	100	85.7	100
*TGFb*1	92.9	92.3	78.6	100	85.7	100
*COMT*	92.9	92.3	71.4	100	85.7	91.7
mean	96.5	96.2	76.8	98.1	89.3	97.9
SD	3.55	3.85	10.6	3.33	6.19	3.59
*GSTM*1	85.7	100	78.6	100	100	100

a Success (%) is the proportion of nail-extracted DNA samples that were successfully genotyped, before and after WGA

b Concordance (%) is the proportion of these successfully genotyped nail-extracted DNA samples that were consistent with those obtained from whole blood DNA.
